# The impact of a dedicated social media strategy on enhancing surgical education

**DOI:** 10.1007/s00423-023-03148-0

**Published:** 2023-10-19

**Authors:** Carly N. Bisset, Frank D. McDermott, Deborah S. Keller

**Affiliations:** 1 Department of General Surgery, Royal Alexandra Hospital, Paisley, PA2 9PN UK; 2 Department of Colorectal Surgery, Royal Devon and Exeter Foundation Trust, Exeter, Devon EX2 5DW UK; 3 Division of Colorectal Surgery, Marks Colorectal Surgical Associates, Lankenau Medical Center, Wynnewood, PA USA

**Keywords:** Social media (SoMe), Twitter, Colorectal surgery, Surgical research, Surgical innovation, Altmetrics

## Abstract

**Purpose:**

Social media (SoMe) is increasingly important in surgical education and may be necessary in the current learning environment. Whilst expanding in use and applications, few studies detail the impact of SoMe on measurable outcomes. The goal of this study was to quantify the impact of a dedicated SoMe strategy on engagement metrics for surgical research.

**Methods:**

A retrospective review of a peer-reviewed surgical journal’s Twitter microblog platform (@ColorectalDis) was performed from 6/2015 to 4/2021. A formal SoMe strategy was introduced in September 2018. Data were stratified into 2 time periods: pre-intervention (6-2015 to 9-2018) and post-SoMe intervention (9-2018 to 4-2021). The main outcome was the impact of the SoMe strategy on user engagement with the Twitter platform, journal, and traditional journal metrics. Twitter Analytics and Twitonomy were used to analyse engagement.

**Results:**

From conception to analysis, the microblog published 1198 original tweets, generating 5 million impressions and 231,000 engagements. Increased account activity (increased tweets published per month—5.51 vs 28.79; *p* < 0.01) was associated with significant engagement growth, including new monthly followers (213 vs 38; *p* < 0.01) and interactions with posted articles (4,096,167 vs 269,152; *p* < 0.01). Article downloads increased twenty-fold post-SoMe intervention (210,449 vs 10,934; *p* < 0.01), with significant increases in traditional journal metrics of new subscribers (+11%), article submissions (+24%), and impact factor (+0.9) (all *p* < 0.01).

**Conclusion:**

SoMe directly impacts traditional journal metrics in surgical research. By examining the patterns of user engagement between SoMe and journal sites, the growing beneficial impact of a structured social media strategy and SoMe as an educational tool is demonstrated.

## Introduction

Social media (SoMe) is a collective term for online-based applications that facilitate seamless connection and communication between users [1]. SoMe offers the unique benefits of free, real-time communications in multiple forms to users at all levels across the world, promoting engagement, equity, and collaboration. As such, SoMe platforms are consistently increasing in use and popularity in general [2]. The same trend is seen in medicine. A recent systematic review of undergraduate medical students demonstrated the ability of SoMe to improve short-term knowledge in both self-reported and objective assessments, flatten hierarchies, and improve communication with educators [3]. In surgery, SoMe offers additional unique opportunities to connect with a larger and more diverse group of investigators to directly interact with patients, colleagues, and the public and effectively recruit for research [4–6]. This has especially proven true in the colorectal surgery community, where investigators have favoured the Twitter microblog platform to disseminate study findings to a global audience, generate discussion with investigators at all experience levels, and increase the visibility of academic work [7, 8]. Members may generate discussion using a mixture of text, visual, and audio media, succinctly delivering the key points of their content; thus, Twitter is an attractive educational resource for trainees and surgeons in practice who need to be time efficient [9].

Whilst increasing in use, little work to date has measured if SoMe increases the reach and impact of surgical research published by journals [10]. Journals traditionally used citation-based bibliometric indices, such as article citations, CiteScore metrics, impact factor (calculated by dividing the total number of citations in a given year for publications in one journal in the preceding 2 years by the total number of citable items published in the preceding 2 years), h-index, and the SCImago Journal Rank, to quantify the journal- and author-level success of a publication. However, these measures are inconsistent in application and interpretation and fail to recognize scholarly activity on social media [11]. The range of metrics used by the scientific publishing community has grown in recent years to consider downloads, reviews, mentions, and shares on social media as legitimate indicators of impact. Alternative metrics (Altmetrics) have emerged that give insight into public impact, predict future citations, and track attention for non-traditional research outputs [12, 13]. For journals, SoMe offers engagement on study findings and implications for clinical practice, facilitates connection with stakeholders, and amplifies published work for increased visibility, article reads, and citation rates, which in turn may influence the impact factor of the journal [14, 15]. Despite the proven value, there is little work to date that quantifies the impact of a journal’s social media strategy, as most work published previously has focussed on the influence of social media on individual article metrics and reach [10, 16]. Determining these journal-specific metrics is important to first establish a baseline and, second, track outcomes for this growing surgical education tool.

The primary aim of this study was to evaluate the impact of a dedicated SoMe strategy on engagement metrics with surgical research published within one international journal—*Colorectal Disease*. Secondly, we aimed to quantify the influence of social media on evidence-based medicine and surgical education. Our hypothesis was that a dedicated SoMe strategy would translate to increased traffic to the journal’s surgical research that was measurable, generalisable, and reproducible.

## Methods

### Study outline

A retrospective review of user engagement metrics for the SoMe microblog platform (Twitter®) for the *Colorectal Disease* journal from June 2015 to April 2021 was performed. Analysis was stratified into 2 time periods: firstly, pre-intervention (Phase 1: June 2015–September 2018, 39 months) and post-intervention (Phase 2: September 2018–April 2021, 32 months), following the implementation of a targeted SoMe strategy. The SoMe strategy consisted of daily posting, consistent branding, and a defined weekly tweet structure, under the leadership of dedicated SoMe editors and team members. User engagement was defined as SoMe and website interactions, journal traffic, and article downloads, which were assessed using Altmetrics, Twitonomy, Twitter Analytics, and univariate analysis. The main outcome measures were to identify changes in user engagement with the journal before and after the implementation of the SoMe strategy.

### Definitions

Twitter Analytics and Twitonomy (Digonomy Pty Ltd) are online analytics tools which monitor and analyse patterns of user engagement with tweets. The definitions used by these SoMe analytical tools are summarised in Table 1.

**Table 1:** Definitions used by Twitter Analytics and Twitonomy

Twitter terminology	Definition
Tweet	A 280-character or less message shared on Twitter, visible to other users
Handle	The username particular to a Twitter account beginning with ‘@’, for example, @ColorectalDis
User	Anyone with a Twitter account including individuals, groups, or institutions
Followers	Number of users who have chosen to view (follow) tweet updates from a particular user
Engagements	Number of times users interact with a tweet, for example—‘liking’ or ‘retweeting’
Impressions	Number of times a tweet is seen by users of the platform
Engagement rate (ER)	ER = [Engagements / Impressions] × 100
Likes	The indication of an individual user’s approval of, support for, or enjoyment of an individual tweet’s content—an example of user engagement with tweets
Retweet	The forwarding of a tweet from another account and sharing the tweet content with users following their own account—an example of user engagement with tweets

### Outcome and exposure

The outcomes are described in 3 parts: firstly, changes in account activity (e.g., differences in the number of tweets posted by @ColorectalDis, total number of impressions); secondly, engagement with followers (including total number of engagements, engagement rates of tweets, and URL clicks); and lastly, account growth (mean number of new account followers per month). New monthly follower data is only available from July 2016 using Twitter Analytics, although the total number of followers from June 2015 to June 2016 is available.

### Statistical analysis

Statistical analysis was performed using descriptive statistics, with frequency (*n*) and percentages (%) for occurrences. Comparative analysis was performed using Student’s paired *t*-tests. Statistical significance was set at an alpha level < 0.05.

### Ethics

There are currently no EQUATOR reporting parameters that address social media metrics. Publication of this work could be a precedent for establishing standardised terms and reporting variables. This study does not use any protected health information and was exempt from institutional review board consideration.

## Results

From 25th June 2015 to the time of writing (30th April 2021), the *Colorectal Disease* Twitter handle has published 1198 organic tweets (excluding retweets without comment), generating almost 5 million impressions and over 230,000 engagements. At the time of writing, the total number of tweets published by the @ColorectalDis handle (inclusive of retweets without comment) was 3409 tweets. Mean monthly comparisons between the 2 phases are demonstrated in Table 2.

**Table 2:** Comparison of mean monthly account activity of @ColorectalDis

Account activity (per month)	Phase 1 (pre-SoMe)	Phase 2 (post-SoMe)
Mean number of tweets	5.51	30.72
Mean number of impressions	269,154	472,1198
Mean number of engagements	10,934	236,369
Mean URL click links	90.59	964.47
Mean new followers	139.68	212.61

### Activity of @ColorectalDis

Significant increases in the total and mean monthly tweets posted before (215 vs 5.51) and after the SoMe strategy (835 vs 28.79; *p* < 0.01) were observed. Similarly, the total number of tweet impressions increased from 269,152 in Phase 1 to 4,721,198 impressions for tweets posted in Phase 2, with an average of 1251.87 impressions per tweet pre-intervention versus 4802.85 impressions per tweet post-intervention. The total number of interactions with published articles was significantly higher post-SoMe than pre-SoMe (4,096,167 vs 269,152; *p* < 0.01 respectively).

### Engagement with @ColorectalDis

Increasing engagement from followers was observed between the 2 phases. The total number of engagements in Phase 1 was 10,934, with an average of 50.86 engagements per tweet, increasing to 236,369 engagements in Phase 2, with an average of 240.46 engagements per tweet (an almost fivefold increase in engagements). The engagement rate in Phase 1 was 4.06%, rising to 5.01% in Phase 2. The total number of URL link clicks relating to published tweets also increased between the 2 phases, with 3533 URL clicks in Phase 1. This increased ninefold to 30,863 URL clicks in Phase 2.

### Account growth and traditional metrics

Trends in increasing activity and engagement were also accompanied by significant differences in the number of followers, with a monthly average of 38 new followers per month in Phase 1 versus 213 (*p* < 0.01) new followers per month in Phase 2. This is also reflected by an increase in new subscribers (up 11%, *p* < 0.01). At the time of writing, the account has 12,283 followers. Article downloads increased nearly 20-fold (210,449 post-SoMe vs 10,934 pre-SoMe; *p* < 0.01). Article submissions increased by 24% during this timeframe (*p* < 0.01), as did the journal’s impact factor by 0.9 (*p* < 0.01).

### Examples of ‘good practice’ used as part of the SoMe strategy

**Fig. 1:**
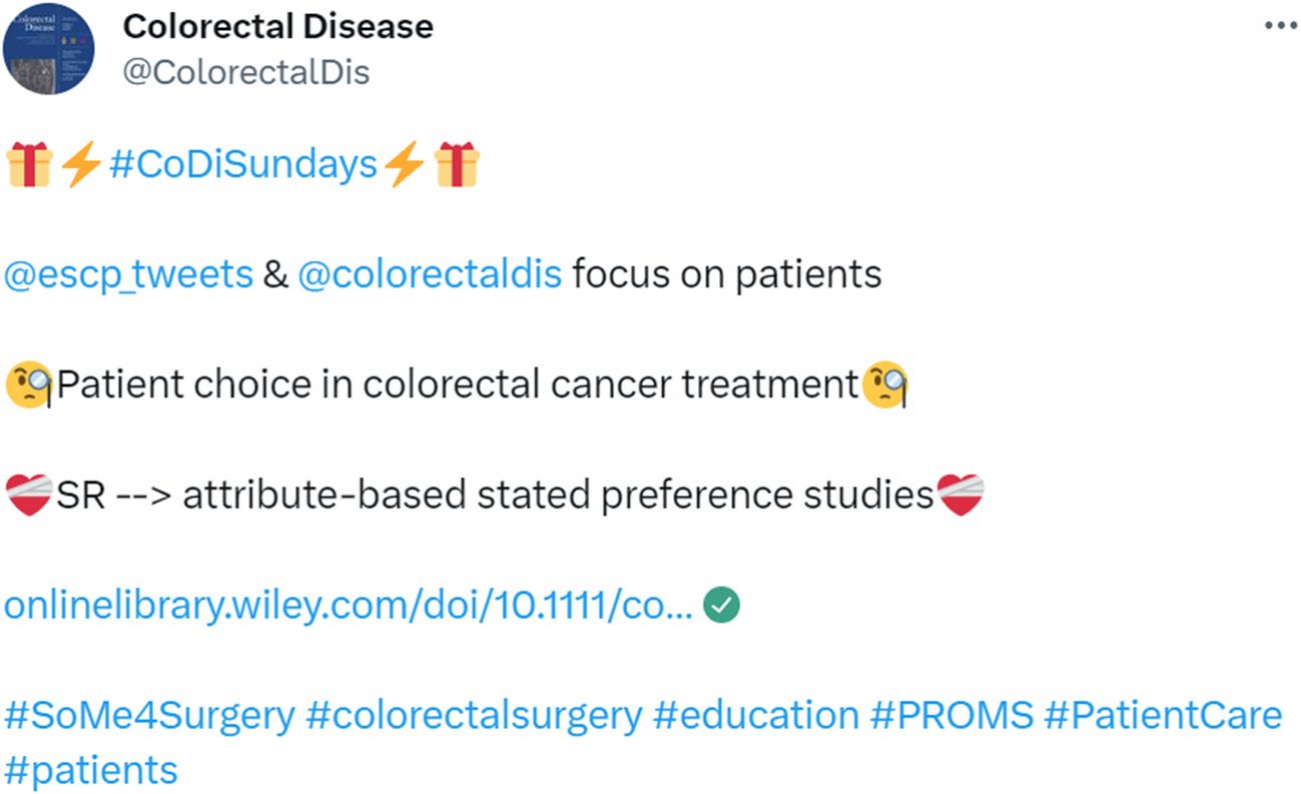
Example of tweet content from @ColorectalDis

**Fig. 2:**
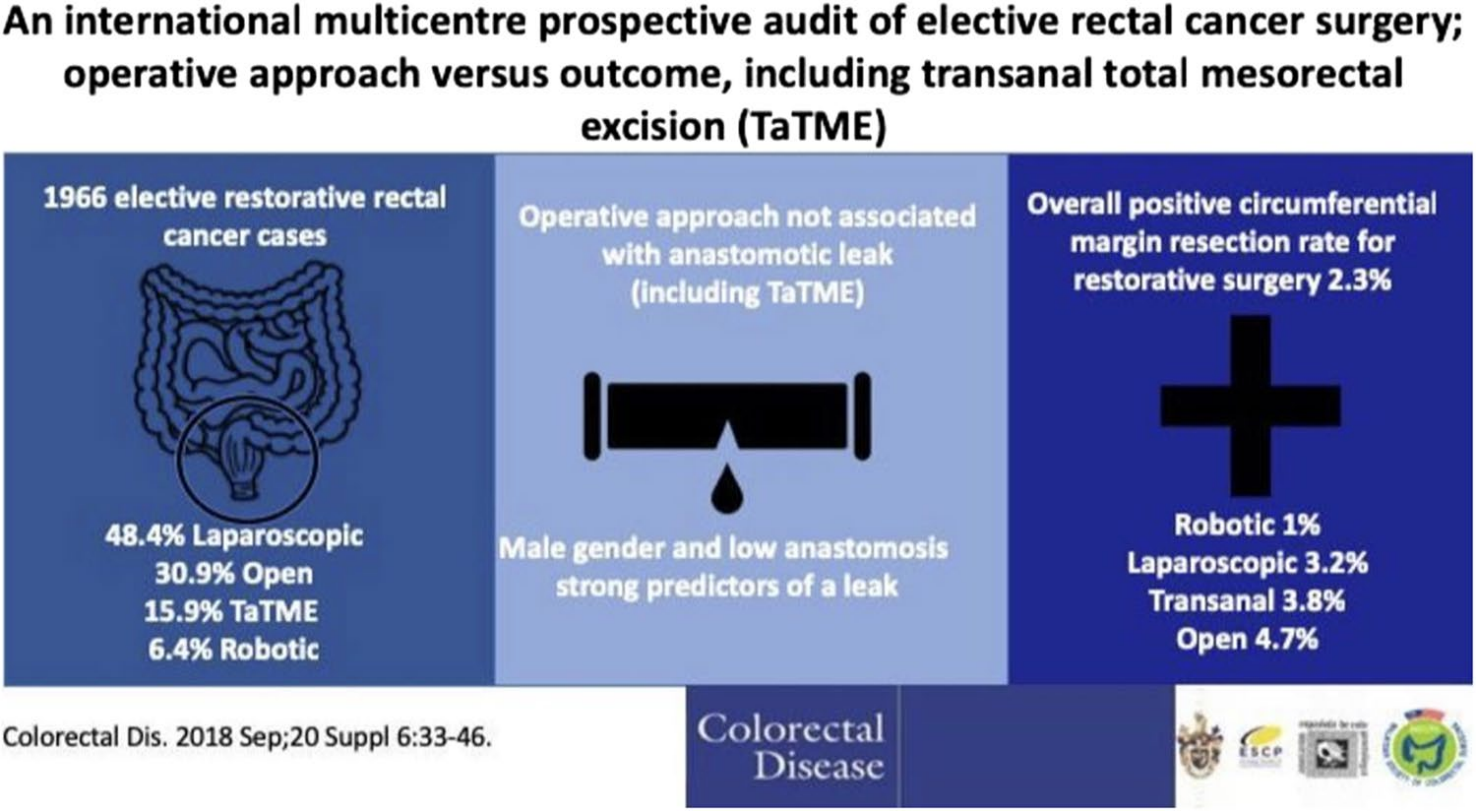
Example of visual abstract from @ColorectalDis

Increased tweet activity corresponded to specific events such as the Association of Coloproctology of Great Britain and Ireland (ACPGBI) Annual Meetings, weekly ‘takeovers’ by six junior editors, and themed ‘tweetchats’ which used the hashtag #colorectaldischat. Some examples of approaches to SoMe engagement used by @ColorectalDis are listed in Table 3 and shown in Figs. 1 and 2.

**Table 3:** Examples of SoMe engagement used by @ColorectalDis

Examples of social media engagement strategies	Explanation of engagement strategy	Likely benefits
Use of hashtag within tweets, e.g., #colorectaldischat and #colorectalresearch	Hashtags allow the grouping of relevant, connected tweets under one ‘umbrella’ term, which may be used in the ‘search’ function of the application	● Focusses tweet content ● Increases viewership as users can search for specific hashtags ● Helpful branding for journal
Weekly ‘takeover’ by junior editor	A designated user given full control of the journal handle (e.g., @ColorectalDis) for a specified period & responsible for content	● Different perspectives and research interests accounted for utilising a variety of experiences within the editorial team ● May increase engagement with new users to the platform, broadening followership ● Workload of managing @ColorectalDis handle spread out
‘12 Days of Christmas’	Each associate editor asked to select their favourite article published in the preceding 12 months with their explanation	● Encourages different focus and perspectives of each editor ● Facilitates inclusion of all types of gastrointestinal research
Putting evidence into practice (#PEIP)	A virtual journal club discussion hosted by ACPGBI, with direct discussion with the study authors on how the implications of their surgical research may be transferred into clinical practice	● **‘**Journal club’ style discussion attractive for engagement ● Encourages ‘Q&A’ style interaction directly with study authors ● Experiences of study authors may influence the clinical implementation of findings and inform future researchers, in turn influencing the journal’s impact
Themed *Colorectal Disease* ‘Tweetchat’ (#colorectaldischat), e.g., ‘ACPGBI Guidelines for Surgery in IBD’	Live 1-h length ‘tweetchat’ (i.e., virtual interview) with a panel of invited colorectal researchers in ‘Q&A’-style	● Engaging tweet content and generation of discussion ● Allows researchers to publicise their work and engage with the colorectal community

## Discussion

This study highlights the advantages that a dedicated social media strategy has for surgical education and journal engagement with its stakeholders. In just 2.5 years, the benefits of a motivated core group without commercial SoMe experience or specific training have rapidly increased the reach of authors’ work within *Colorectal Disease* and increased their presence within the colorectal surgery community. For the foreseeable future, a designated multimodal SoMe approach is likely to remain important in maintaining journal relevance and being representative of and of interest to the journal’s readership.

Whilst Twitter is not necessarily the most popular global social media platform in terms of crude user numbers, the platform and its interface have a well-established clinician and patient base [17, 18]. Although Facebook is the most popular platform, with an estimated 1.7 billion users worldwide [19], surgeons appear to prefer the use of Twitter as a professional networking and microblogging tool. Facebook is often seen as more ‘personal’, with clinicians preferring to use this platform for connecting with friends and family due to enhanced privacy settings, compared to other more public platforms such as Twitter or Doximity [20]. Whilst ‘closed groups’ exist, the uptake by the medical community for using Facebook other than for advertising reasons is limited [21]. Instagram (owned by Facebook) is another popular platform which is primarily a photo-blogging application. This platform is traditionally more popular with younger demographics for image and video-sharing content, with an estimated 631 million users aged between 18 and 34 years old and 995 photos posted every second [22]. However, it is an application that is primarily for personal use or product advertisement, with seemingly limited engagement at present with surgical academia [23]. It may be that journals can utilise applications like Instagram as part of their SoMe educational strategy in the future, particularly to widen participation amongst medical students and surgeons-in-training (for example, using visual abstracts and reels) [24, 25]. Interestingly, there is evidence to show that the use of visual abstracts increased the number of impressions by 7.7-fold and retweets by 8.4-fold compared to tweets containing article titles alone [26]. It is therefore important that a journal’s SoMe team is forward-thinking and considers the future use of other SoMe platforms, as well as keeping abreast of its own monthly Twitter metrics to monitor trends in user engagement.

With huge increases in the @ColorectalDis follower base and higher engagement rates, the journal may be considered an ‘influencer’ account within colorectal academia [27]. ‘Influencers’ are generally considered to be SoMe accounts which ‘disproportionately impact the spread of information or some related behaviour of interest’ [28]. Thus, influence works on the principle of homophily, which inevitably may create echo chambers. Interestingly, one study using statistical modelling on Twitter demonstrated that the ‘word-of-mouth’ spread of small cascades of information is more likely to result in greater reach than information distribution from one very large following account [28]. Therefore, whilst a large follower count is a crude reflection of popularity and interest, individual users (such as engagement from journal editors, authors, and clinicians) are necessary to maximise information dissemination. It is currently unclear what the ‘target’ number of followers is required (the ‘number needed to tweet’); however, in comparison to other leading surgical journals, those with the highest impact factor tend to have a large follower base, regular tweet updates, use visual abstracts, and have a designated SoMe editor [29]. It is also unclear if it is the journal’s high impact factor itself which drives its popularity and engagement, if social media engagement increases the influence and thus impact factor of the journal, or if this is a mutually beneficial relationship. We hypothesise that it is likely to be the latter.

As with all SoMe platforms, caution must be exercised with their usage. Whilst *Colorectal Disease* is a peer-reviewed journal, caution must be considered regarding tweet content, as it is a digital and public representation of the journal. This places increased responsibility on those managing professional and educational SoMe accounts to post responsibly. Guidance on the ethical usage of SoMe in the UK is provided by the GMC [30], which also highlights the importance of respectful interactions between users. Careful consideration of tweet content is necessary to prevent the propagation of ‘fake news’, i.e., the spread of misinformation from sources of varying credibility and transparency [31, 32]. This may be exacerbated through an ‘echo chamber’ effect, where social media feeds are often tailored to display posts from like-minded users who share similar beliefs which further amplifies misinformation [33]. Other considerations for responsible SoMe usage include the potential for a security breach or an unauthorised user obtaining access to the account. Regular changes of passwords limited to a core group of SoMe team members may aid data security [34]. A further drawback of SoMe (particularly for content creators) is the increased risk of burnout associated with high levels of ‘screentime’, which may make it difficult for researchers to fully ‘switch off’ outside of work and on annual leave [35, 36].

Limitations within this study include an inherent English-language bias. Whilst efforts are made for the journal to be of interest to a global audience, the distribution map of followers has clearly demonstrated a predisposition to English-speaking countries, despite the increasing presence of Spanish-speaking (amongst others) surgeons using Twitter [37]. A distribution map was generated using the geo-locations inputted by followers as part of their account biographies (encompassing only one-fifth of the total number of @ColorectalDis followers (*n* = 2270)); therefore, the map may not necessarily be a true representation of where @ColorectalDis followers are based. Whilst the editorial committee (including junior editors) encompasses a wide variety of surgeons and surgeons-in-training from across the world with differing subspecialty interests, there is always room for increasing diversity of thought and collaboration which will in turn reduce the likelihood of an ‘echo chamber’ effect. A further limitation is that the impact factor of a journal may also be influenced by the publication of articles of greater interest to the wider public and readership or by greater overall numbers of submissions to journals. With the wider adoption of social media, other surgical Twitter accounts have seen an increase in followers. However, we did not have access to other surgical accounts to assess their metrics. It is not possible to confirm the causality of whether the increase in @ColorectalDis metrics was due to the new strategy or due to the increasing popularity and engagement with #SoMe, including other social media platforms.

## Conclusion

The relationship between surgical academia and social media has changed drastically in the last 10 years. Guideline updates, surgical innovation, the sharing of ideas, and networking amongst global peers are now only ‘clicks’ (or tweets) away. For journals to remain relevant to their readership, a designated social media strategy is necessary. With increasing popularity, Twitter remains the ideal SoMe platform for journals to engage regularly with their stakeholders, and SoMe is likely to bear increasing importance in determining and influencing a journal’s impact factor. This study has demonstrated that a dedicated SoMe strategy has increased access to the journal’s content, increased user engagement and number of article downloads, and increased traffic to the journal’s website, providing further evidence that SoMe activity may advance surgical research and education, thus leading to a paradigm shift towards alternative academic metrics.
